# Adoption of Telemedicine in a Rural US Cancer Center Amid the COVID-19 Pandemic: Qualitative Study

**DOI:** 10.2196/33768

**Published:** 2022-08-16

**Authors:** Matthew Mackwood, Rebecca Butcher, Danielle Vaclavik, Jennifer A Alford-Teaster, Kevin M Curtis, Mary Lowry, Tor D Tosteson, Wenyan Zhao, Anna N A Tosteson

**Affiliations:** 1 Department of Community and Family Medicine Geisel School of Medicine at Dartmouth Lebanon, NH United States; 2 The Dartmouth Institute for Health Policy & Clinical Practice Geisel School of Medicine at Dartmouth Lebanon, NH United States; 3 Connected Care Dartmouth Health Lebanon, NH United States; 4 Center for Program Design and Evaluation Geisel School of Medicine at Dartmouth Lebanon, NH United States; 5 Dartmouth Cancer Center Dartmouth Health Lebanon, NH United States; 6 Department of Biomedical Data Science Geisel School of Medicine at Dartmouth Lebanon, NH United States; 7 Department of Medicine Geisel School of Medicine at Dartmouth Lebanon, NH United States

**Keywords:** telemedicine, telehealth, remote consultation, clinical oncology, implementation science, qualitative research, telemedicine methods, telemedicine organization and administration, telemedicine trends, clinical oncology methods, clinical oncology organization and administration, oncology, digital health, virtual care, COVID-19

## Abstract

**Background:**

The COVID-19 pandemic necessitated a rapid shift to telemedicine to minimize patient and provider exposure risks. While telemedicine has been used in a variety of primary and specialty care settings for many years, it has been slow to be adopted in oncology care. Health care provider and administrator perspectives on factors affecting telemedicine use in oncology settings are not well understood, and the conditions associated with the COVID-19 pandemic offered the opportunity to study the adoption of telemedicine and the resulting provider and staff perspectives on its use.

**Objective:**

The aim of this paper is to study the factors that influenced telemedicine uptake and sustained use in outpatient oncology clinics at a US cancer center to inform future telemedicine practices.

**Methods:**

We used purposive sampling to recruit a mix of oncology specialty providers, practice managers, as well as nursing and administrative staff representing 5 outpatient oncology clinics affiliated with the Dartmouth Cancer Center, a large regional cancer center in the northeast of United States, to participate in semistructured interviews conducted over 6 weeks in spring 2021. The interview guide was informed by the 5 domains of the Consolidated Framework for Implementation Research, which include inner and outer setting factors, characteristics of the intervention (ie, telemedicine modality), individual-level factors (eg, provider and patient characteristics), and implementation processes. In total, 11 providers, 3 leaders, and 6 staff participated following verbal consent, and thematic saturation was reached across the full sample. We used a mixed deductive and inductive qualitative analysis approach to study the main influences on telemedicine uptake, implementation, and sustainability during the first year of the COVID-19 pandemic across the 5 settings.

**Results:**

The predominant influencers of telemedicine adoption in this study were individual provider experiences and assumptions about patient preference and accessibility. Providers’ early telemedicine experiences, especially if negative, influenced preferences for telephone over video and affected sustained use. Telemedicine was most favorably viewed for lower-acuity cancer care, visits less dependent on physical exam, and for patient and caregiver education. A lack of clinical champions, leadership guidance, and vision hindered the implementation of standardized practices and were cited as essential for telemedicine sustainability. Respondents expressed anxiety about sustaining telemedicine use if reimbursements for telephonic visits diminished or ceased. Opportunities to enhance future efforts include a need to provide additional guidance supporting telemedicine use cases and evidence of effectiveness in oncology care and to address provider concerns with communication quality.

**Conclusions:**

In a setting of decentralized care processes, early challenges in telemedicine implementation had an outsized impact on the nature and amount of sustained use. Proactively designed telemedicine care processes with attention to patient needs will be essential to support a sustained role for telemedicine in cancer care.

## Introduction

The COVID-19 pandemic led to an unprecedented need to deliver care for cancer and other conditions remotely [1-4]. Telemedicine has long been touted as a promising but underused mode of delivering cancer care, especially in rural areas where access is often constrained by the need to travel significant distances [5-12]. While technologies to support telemedicine have been around for decades [13-15], it was only when the public health emergency occurred locally—necessitating the curtailing of all nonessential in-person contact in March 2020 [16-21]—that our region in the rural New England region of the United States experienced a rapid uptake. At the start of the pandemic, telemedicine support at the Dartmouth Cancer Center (DCC) was provided by a small department used to handling a fraction of the visits experienced during the pandemic (outpatient televisit rates increased some 10,000%). The basic visit process entailed a multistep setup requiring the patient to download, install, and configure a computer software or smartphone app, or to be available for a phone call for a telephone visit once payment policy shifted to permit telephone visits [16-20,22]. After the first 3 months of the pandemic, the video visit process simplified to one where video visits could occur via a much simpler application accessible via the patient portal either on a computer or smartphone. Resources supporting the transition to televisits were largely limited to web-based training materials for learning to use the telemedicine platform, without the capacity for providing technical support or individualized workflow adaptations at the department or clinic level.

Quantitative analysis of telemedicine use (including the use of either telephone or video to provide real-time care similar to an in-person office visit) within the DCC over a 1-year time frame from pandemic onset revealed relatively low use compared to other specialties [23], and further analysis showed significant variability in use by clinic site corresponding to a larger magnitude of difference in telemedicine use rates compared with patient, geographic, or medical factors [24].

In a broader context, published studies of use trends of telehealth for cancer care suggest disparities in telehealth use, with patients in urban settings favoring telehealth more than rural [25], as well as other groups including older adults and patients of color [26]. Recent qualitative studies of telehealth for cancer care during the COVID-19 pandemic suggest that there is a subset of care situations within survivorship that is acceptable to providers and patients alike [27], and that telehealth has broadly been acceptable to many patients and providers even as concerns about a lack of physical exam are raised [28].

To better understand the underlying factors to the observed local variation in use amid the rapid transition in care delivery, we conducted a rapid-cycle qualitative study of semistructured interviews with a diverse mix of oncology providers and clinic staff in the spring of 2020.

## Methods

### Study Setting

The DCC, an affiliate of Dartmouth Health, serves the bistate region of New Hampshire and Vermont as well as parts of New York, Massachusetts, and Maine with headquarters at Dartmouth-Hitchcock Medical Center (DHMC) in Lebanon, New Hampshire. DCC operates 5 oncology clinics serving 18,000 to 20,000 patients per year across the catchment area. The proportion of patients who are dual eligible for both Medicaid (state-sponsored insurance for eligible low-income patients) and Medicare (nationally sponsored insurance for eligible older adults aged 65 or older or with specific disabilities) ranges from 19.1% to 25% across the oncology clinic sites (Table 1).

Approximately 71% of patients seen in 2020 resided in rural settings [29]. The COVID-19 pandemic impacted care starting in mid-March 2020, with Dartmouth-Hitchcock Medical Center responding to a state-mandated lockdown by postponing or transitioning all nonessential care to telemedicine on March 18, 2020. Restrictions continued until April 30, 2020, at which point efforts sought to normalize care volumes through screening processes and visitor restrictions while continuing to incorporate telemedicine where appropriate.

**Table 1:** Patient demographic characteristics as a percentage of total population served by DCC^a^ across oncology clinics in 2019.

Characteristics		Clinics				
		A (n=2341), n (%)	B (n=9923), n (%)	C (n=2631), n (%)	D (n=1882), n (%)	E (n=1502), n (%)
**Race**						
	White	2271 (97.0)	97.3 (9655)	2394 (91.0)	1673 (88.9)	1458 (97.1)
	Black	12 (0.5)	60 (0.6)	84 (3.2)	47 (2.5)	6 (0.4)
	Hispanic	14 (0.6)	99 (1.0)	84 (3.2)	85 (4.5)	11 (0.7)
**Sex**						
	Female	1367 (58.4)	5487 (55.3)	1584 (60.2)	1150 (61.1)	765 (50.9)
	Male	974 (41.6)	4436 (44.7)	1047 (39.8)	732 (38.9)	737 (49.1)
Medicaid^b^		447 (19.1)	1935 (19.5)	658 (25.0)	356 (18.9)	360 (24.0)
**Age**						
	>65	1470 (62.8)	5517 (55.6)	1181 (44.9)	804 (42.7)	993 (66.1)
	>85	206 (8.8)	506 (5.1)	134 (5.1)	83 (4.4)	93 (6.2)

^a^DCC: Dartmouth Cancer Center.

^b^Includes those *dual eligible* for Medicaid and Medicare.

### Sampling and Recruitment

Across the 5 clinic locations, we recruited a purposive sample of oncology clinical providers, leaders, regional practice managers overseeing telemedicine implementation, and nonphysician staff (eg, schedulers and nurse managers) to participate in semistructured interviews. Of the 67 medical doctors and nurse practitioners employed at DCC who used some amount of telemedicine between January 2020 and October 2020, we sampled 30 clinical providers representing low-to-high telemedicine use, a mix of oncology specialties, the 5 clinic locations, and varied proportion of rural patients served. Leaders, practice managers, and clinical providers were recruited through direct email invitations from study leaders. Following interviews with regional managers, we used snowball sampling to identify a mix of other nonphysician staff members representing all 5 clinics with direct experience supporting telemedicine during the pandemic.

### Data Collection and Analysis

We used the Consolidated Framework for Implementation Research (CFIR) to inform data collection and analysis. The CFIR includes over 30 evidence-based constructs grouped within the 5 domains of intervention characteristics, outer setting, inner setting, characteristics of individuals targeted by the intervention, and the process of implementation. The CFIR was developed to examine complex interventions across different settings, including hospitals as well as primary care and telehealth settings [30-34]. We created 2 semistructured interview guides for providers and staff, which explored constructs from all 5 CFIR domains with particular emphasis on identifying barriers and facilitators to telemedicine uptake and sustainability associated with inner or outer contextual factors; telemedicine technology and functionality; provider experiences, knowledge, and attitudes toward telemedicine technology given typical clinical workflows; perceptions of patient and caregiver attitudes and capabilities in using the technology; and overall implementation processes and adaptability. Following the creation of the guides and use in a few initial interviews, we made final modifications to the question wording and probes to improve interview clarity, flow, and consistency among the interviewers.

Two researchers (DV and RB) conducted semistructured interviews with cancer care providers, and 1 researcher (JAT) conducted all staff interviews. None of the interviewers had explicit clinical experience or roles within the institution, and all of them were unknown to interview participants. All 3 researchers listened to a sample of the early interviews and then met to debrief, adjust the interview guide as noted above, and reach consensus on interview methods before completing future interviews. The interviews lasted approximately 45 minutes and were recorded with participant permission for later transcription.

We used the web-based transcription service, “Rev.com,” to create word-for-word transcripts of the interviews that were then uploaded into the qualitative analysis platform Dedoose (Socio Cultural Research Consultants, LLC). Two researchers (DV and RB) coded and analyzed the transcripts using mixed inductive and deductive methods [35,36]. The 2 researchers coded a sample of transcripts separately and then met to debrief and reach consensus. The researchers used a mix of in-person debriefing sessions, emails, and internal Dedoose memos to reach consensus and discuss any personal or professional biases that arose throughout the analysis process. If the coding team was unable to reach consensus, additional members of the study team were consulted (JAT, MM, and AT). Thematic saturation was reached across the full sample as evidenced by no new themes and subthemes coming forth in exploring the main CFIR constructs of interest [37].

### Ethical Considerations

The study received Dartmouth Health institutional review board approval (STUDY 02000578). The participants were provided with an information sheet and opportunity to ask questions prior to participation. The participants were verbally consented, and permission was obtained for the audio recording of interviews. Names, titles, and practice settings were deidentified and will not be included in any published data.

## Results

### Overview

We conducted 11/20 (55%) provider, 3/20 (15%) leader, and 6/20 (30%) staff interviews (Table 2). All providers reported adopting some form of telemedicine for a significant proportion of visits in the early months of the pandemic (March-June 2020), predominantly via phone (vs video), consistent with our prior quantitative analysis [24]. Telemedicine use (video and phone combined) by the providers in this sample also mirrored that of the entire DCC oncology service, which peaked at an average weekly telehealth visit rate of 26% of visits in the initial lockdown phase, settling out to an average of 10%-12% after lockdown [23].

**Figure 1:**
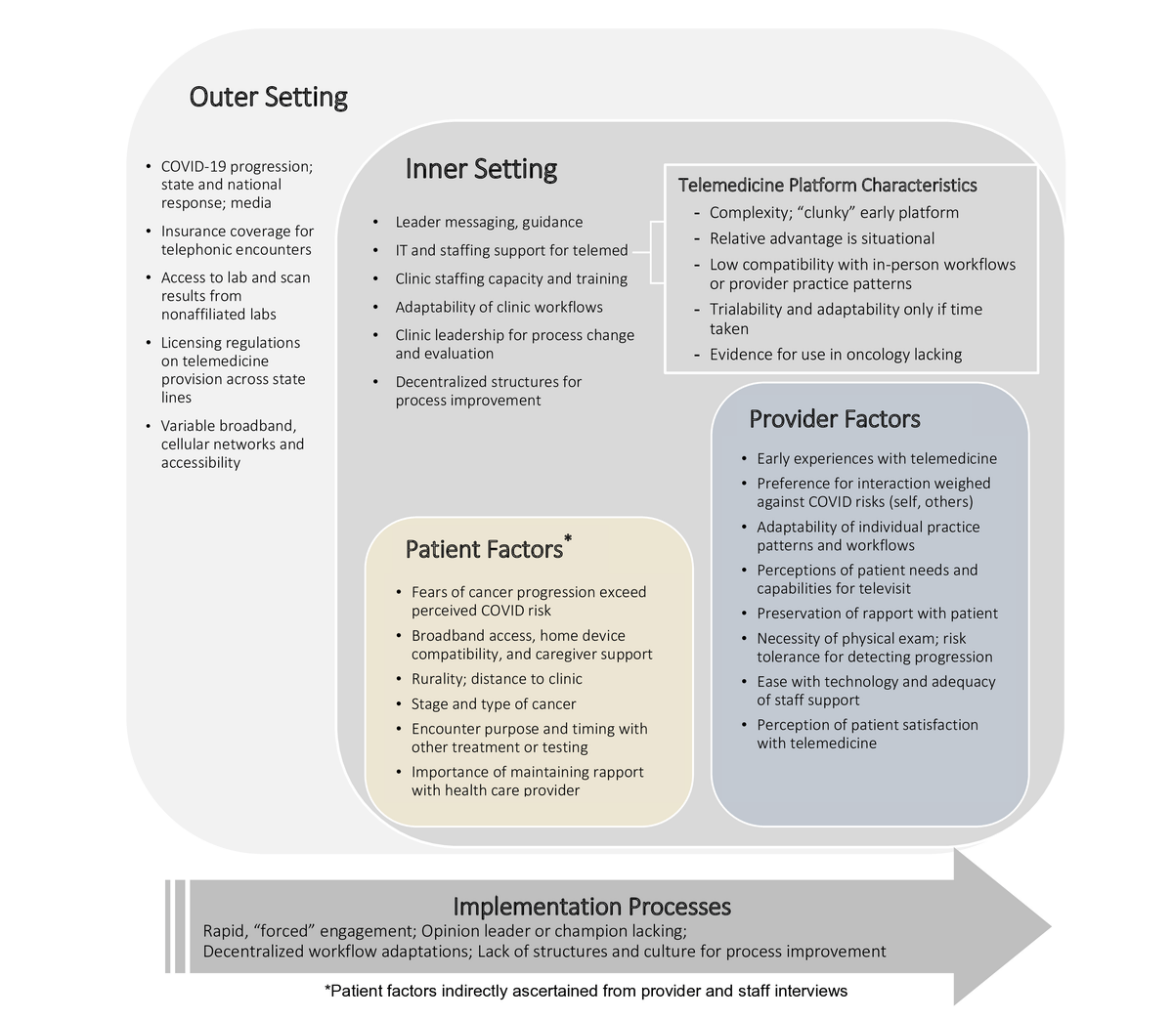
Main themes organized by the Consolidated Framework for Implementation Research construct; telemed: telemedicine.

**Figure 2:**
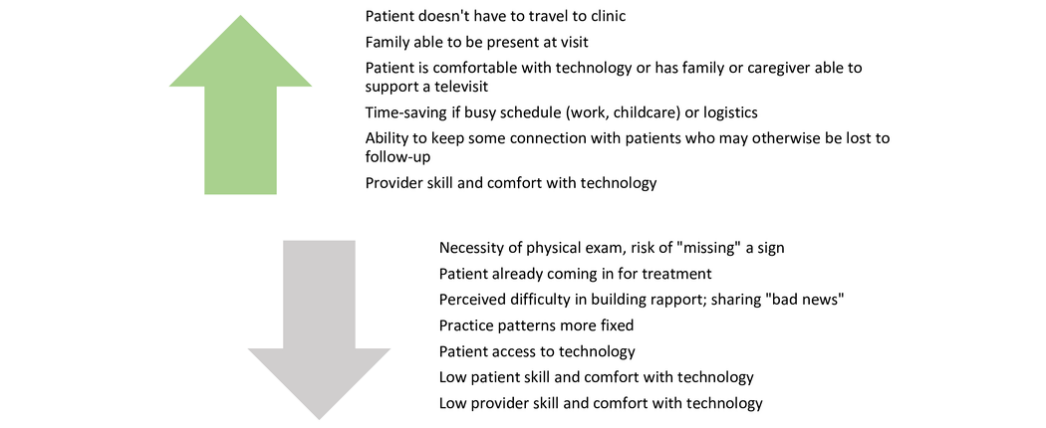
Facilitators and barriers to telemedicine uptake.

The qualitative findings shed light on these use patterns. Figure 1 presents the factors having the greatest influence on uptake and sustained use of telemedicine that emerged in the staff and provider narratives by the CFIR domain. Figure 2 presents a summary of the factors across all CFIR domains, which emerged as either facilitating telemedicine use or acting as barriers dissuading or constraining telemedicine use. The results are summarized below; select quotations can be found in Table 3.

**Table 2:** Interview participant demographics.

Category		Value, n (%)
**Position level**		
	Provider (medical doctors and nurse practitioners)	11 (55)
	Leaders or practice managers	3 (15)
	Staff (schedulers and registered nurse managers)	6 (30)
**Provider telemedicine use**		
	Low (0%-9%)	3 (28)
	Medium (10%-17%)	4 (36)
	High (18%-30%)	4 (36)
**Years in role**		
	Less than 1	5 (25)
	1-4	5 (25)
	5-10	3 (15)
	11-15	2 (10)
	>15	4 (20)
	Not specified	1 (5)

**Table 3:** Selected quotations organized by the Consolidated Framework for Implementation Research (CFIR) domain.

CFIR domain	Exemplar quotes
Outer setting	“The biggest barrier [was ordering and receiving] labs [from other facilities] and putting them into the patient's chart for the doctor to review before the visit… There have been a couple instances where we have had to delay telemedicine visits for our patients because the doctor doesn't have the results yet” (staff). “Well, institutionally, if it's not paid for, they won't use it…They haven't embraced it before COVID. I think COVID just pushed it into the mainstream. So that's a huge barrier in theory. And I hope [insurances] will recognize the benefit” (provider).
Inner setting	“The other piece that we're still trying to create a better workflow for is the rooming process… [Providers] don't have the clinical support to be able to have somebody touch base with the patient prior to them joining the video, or the telephone call if that's the case. That piece is something that we're still trying to make more efficient, is having that virtual rooming where the MA is able to start the visit, collect that information, get it entered into the encounter for the provider” (staff). “The main workflow in the clinic is we get our schedule, and we looked at the schedule, and I go through the patients and look at the diagnosis. And then I decide which patient is appropriate for telehealth, and which patient is not. And it is the secretaries who called the patient” (provider).
Intervention characteristics	“I think the lack of a real physical examination is a real rapport problem. There's something quite unique about the physician doing a physical exam that communicates a lot of unspoken things to the patient… [It is] hard to describe” (provider). “[My mix of phone vs video is three fourths phone and one fourth video]. The videos can be a little tedious and for the video, the patient has an appointment for which they're sitting in front of their computer. So, if I'm running 30-minutes late, they're stuck in front of their computer. Where the phone…they could more or less live their life and go about their day and I'll call them on their cell phone. And so, from my perspective [it] is much more convenient” (provider). “I do think over the telephone I miss the non-verbal cues. If I'm in the exam room or virtually I'm with the patient and a family member and I say something and they get this look on their face and I can say so, you look like you're maybe not comfortable with that or your wife just shook her head in the opposite direction of you. There's more non-verbal cues that then tell me to sort of pursue that a little further, especially things like depression. Sometimes I can tell that more. They may not say they're depressed, but I can tell they're not really maintaining eye contact well or they're kind of a flat affect. You don't get that over the telephone” (provider).
Characteristics of individuals	“For our patients, who are either frail, rural, or both, when there's travel issues, or even they don't have a lot of gas money, like being able to say, “Listen, it's okay. We'll do a tele-visit,” is awesome” (provider). “We have an older, sicker population who may be less computer savvy, may have less access to high-speed Internet, and have a reluctance to incorporate the technology into their lives” (provider).
Implementation processes	“[Providers are using telemedicine] for long-term follow-ups, for discussion visits, for chemo teachings, for results discussion visits, for patients who live far away and don't want to come in. And for patient side, I see it as for the exact same reason, for patients who say, “I live two hours away. Can this be a phone visit?” And we say, “No problem. Happy to help” (staff). “[The providers] see a lot of patients on treatment, so they see them, when they're getting their treatment in the infusion suite… I don't know how many of them have done [telemedicine] in between. I'm just starting to see because we share a lot of patients where they'll do an in between checkup visit by video. I'm seeing a little more of that where they're not actually getting their chemo, but I think the majority of their patients are actually getting treatment the same day they see the provider” (provider leader).

### Outer Setting Factors

The pandemic and the associated public health response by national, state, and institutional leaders were both a trigger for implementing telemedicine and a source for widespread disruption in usual clinical practice workflows. All interview participants described a considerable amount of initial confusion in the transition to telemedicine due to questions about reimbursement allowances (eg, whether Medicaid, Medicare, or commercial insurances covered telephone as well as video visits, and whether payment rates would be the same as in-person care) and provider licensing regulations, compounded by mixed media messages and unknowns related to the spread and exposure of the virus. Another challenge to the early transition reported mainly by staff participants was associated with the shift by many patients to using local, nonaffiliated clinics for lab testing, which were not linked to the electronic health record. Providers and staff alike reported this necessitated additional staff time to obtain and integrate results into the record for providers to have during a telemedicine visit; if it was not obtained, it caused scheduling disruptions. Another external setting factor that emerged was the major policy change allowing reimbursement of telephonic visits at rates on par with in-person or video-based visits. This policy change was a key factor to overcoming technology frustrations experienced in early video visits and was cited by most respondents, providers, and staff alike, as it is important for the sustained use of telemedicine in oncology.

### Inner Setting Factors

According to the participants, practice and provider workflows for using telemedicine were nonexistent at all oncology clinics at the start of the pandemic. Existing clinic workflows for in-person visits were reported to be largely incompatible with the new flows needed for the telehealth transition, and staff and providers alike wished for greater guidance from DCC leaders to help with initial implementation. As noted above, there was considerable confusion and questions about which visits could and could not be carried out via telemedicine in the early days, and the respondents overall reported a lack of clear direction or support from an internal champion to address questions. Moreover, the providers reported that the overall pace of care did not allow for dedicated time to effectively engage with training materials on their own, and there was no institution-wide push to ensure all providers complete telemedicine training.

Clinic leaders, schedulers, and providers reported taking matters into their own hands to develop ad hoc strategies to make the shift to telemedicine early on. Workflows and staff responsibilities were modified to support telemedicine visits. On some teams, staff were tasked with calling patients in advance to prepare them for the telemedicine visit, practice with the technology, and gather medication and medical history information; however, clinics rarely had the staffing resources to carry this out consistently. As the pandemic evolved, clinic teams continued to refine internal workflows, patient messaging, and coordination with new lab vendors to support telemedicine use, all with a high degree of variation across clinics and largely based on local preferences of providers and perceived patient needs.

### Intervention (Telemedicine) Characteristics

Provider dissatisfaction with the telemedicine user interface, particularly with video visits in the early days of the pandemic, emerged as a critical variable in determining ultimate use of the technology for patient care and preferences for phone over video. The providers described the telemedicine platform in use at the onset of the pandemic as “clunky” and requiring multiple steps to log in. Many reported quickly transitioning to phone visits because of the technical challenges both they and their patients encountered with the video interface, citing frustration and wasted time trying to establish and maintain a successful video connection. Even after a year, a few providers in our sample had still not conducted a video visit after hearing about colleagues’ experiences. Following an institutional switch to a different telemedicine platform in the summer of 2020, the participants reported improved connectivity and visit satisfaction, although not enough to convince those more hesitant with the technology to reattempt video visits.

Telemedicine was perceived as holding relative advantage over in-person visits for some clinical situations described below. Moreover, the technology offered providers time savings and greater flexibility in scheduling visits around research, meetings, and serving multiple clinic sites, while it was also reported as reducing travel demands for rural patients and those with busy work, home, and school schedules. Despite these advantages, respondents wanted more evidence of the efficacy of telemedicine, particularly in the context of cancer care where many feared missing disease progression when conducting clinical exams virtually.

Trialability and adaptability with the telemedicine technology happened to varying degrees among clinic teams as reported in the interviews. Novel uses for telemedicine in oncology care emerged during the implementation, most notably in the form of what was locally referred to as “chemo teaches” (meetings to prepare patients and caregivers on what to expect while undergoing chemotherapy) and other patient or family education. Telemedicine allowed family members who were geographically remote or working to participate in education sessions and visits. The easing of state licensure restrictions (also an external setting factor) enabled several providers to provide telemedicine consultation to patients outside of their usual geographic area, supporting continuity for patients who needed to travel as well as enabling new consultations and second opinions.

### Characteristics of Individuals

#### Provider-Level Characteristics and Preferences

Of all the factors influencing telemedicine uptake and implementation, provider preference had the greatest effect on both the ratio of telemedicine to in-person visits and the modality of those telemedicine visits (phone vs video). A combination of early negative experiences with video, comfort with technology (or lack thereof), convenience, and perceptions of patient preferences contributed to a majority of providers in our sample, almost exclusively opting for telephonic visits if in-person visits were not possible.

Preferences were also influenced by attitudes around risk of COVID-19 exposure (self, staff, and patients) balanced against the degree to which providers valued direct patient interactions to connect with patients and assess clinical conditions. Provider willingness to experiment with the technology and adapt individual practice workflows was more of a predictor of telemedicine use than clinical specialty or years in practice (ie, provider age).

Most providers in our sample felt it was harder to achieve their preferred level of rapport with their patients in televisits (phone or video), though some found video visits afforded new ways to connect with patients by observing them in home settings and family encounters. For difficult conversations or when health literacy was in question, in-person and video visits were universally preferred. In the narratives, providers often couched their own preferences around supporting their patients’ preferences (real or perceived). The providers reported using patient preferences to determine visit type yet acknowledged that patient willingness to use telemedicine (either telephonic or video) could be modified by messaging about the different options during appointment scheduling.

#### Perceptions of Patient-Level Characteristics and Preferences

A patient’s geographic distance to the medical center had a mixed effect on telemedicine use. According to staff and providers, for some rural patients, telemedicine offered a solution against frequent, lengthy trips into the clinic for more routine visits (especially in poor weather conditions or when transportation assistance was needed). For other patients, the providers cited reports of poor internet connectivity or cell service, which hindered telemedicine use. The participants gave examples that suggested they would assess a patient’s skill or comfort with technology in determining whether to offer a telemedicine visit. Older, rural patients were reported to be more likely to choose phone or in-person visits rather than using telemedicine technology because of a lack of familiarity with technology. Family or caregiver support (eg, in assisted living settings) was observed by staff and providers to buffer against technology challenges. Younger patients were cited as being more willing to engage with technology but were constrained by other factors, including busy work and family schedules that led providers to offer telephonic visits more often than video visits.

Staff and providers agreed regarding the clinical situations better suited for telemedicine. These included patients with less aggressive or more stable cancers such as hematological cancers; cancers for which a physical exam was less important because scans or lab results largely dictated treatment decisions; patients in remission; or clinical situations where visits could reasonably alternate between in-person and telemedicine (eg, if the patient needed monthly monitoring). These considerations are summarized in Table 4.

**Table 4:** Situations in cancer care better and worse suited to telemedicine use, as reported from staff and provider interviews.

Category	Better for telemedicine	Worse for telemedicine
Cancer type	Less aggressive Generally stable over time (eg, hematological cancers) Monitoring or treatment largely based on labs or imaging scans	Rapidly progressing or unstable Need to assess tolerance to new therapy Physical exam important to assess (eg, breast, GI^a^ or GU^b^, and head or neck cancer)
Visit type	Routine interval monitoring between treatments Patient or family education (eg, chemo teaches) Survivorship follow-up visits	Patient already on site for treatment visit or scan ”Decision point“ for changes (eg, hospice transition or continuation of therapy)

^a^GI: gastrointestinal.

^b^GU: genitourinary.

For more rapidly progressing cancers such as breast, gastrointestinal or genitourinary, as well as head and neck cancers, the providers had a strong preference for seeing their patients in person, as they were concerned they may miss important disease progression that could influence treatment decisions. In these cases, they reported a heavy reliance on the physical exam and other aspects of an in-person visit to assess a patient’s response to and tolerance of treatment, especially around important decision points in care.

Overall, providers and staff reported that while telemedicine can be incorporated into oncology care, the nature of oncology and the fact that patients with oncology-related needs are already coming in for treatment do not lend themselves to a high level of telemedicine adoption. Patient and provider perceptions of confidentiality and privacy concerns in using technology did not emerge as a main theme in this study.

### Implementation Processes

Interview participants voiced a desire for a clear vision for telemedicine use in oncology, substantiated by evidence, supported by recognized champions, and standardized through official policies such as continued reimbursement for telephonic visits in specific clinical situations.

Logistical and workflow improvement recommendations included staff support to virtual “room” patients at the start of a telemedicine visit, establishing dedicated space for televisits, where equipment was already set up, establishing preappointment protocols and scheduling processes to ensure patients were adequately prepared, and clarifying roles and training to ensure clinics had the capacity to support both in-person and televisits in a smooth fashion. Challenges and burdens of staff time in obtaining lab results from outside vendors indicate a need for formal partnerships, data sharing agreements, and integrated electronic systems to share results more efficiently.

The participants identified a need to continue to improve accessibility and ease in using the telemedicine technology for patients and providers alike. Translation services were a challenge for some to incorporate within telemedicine visits. The providers voiced a need for more training and peer-to-peer learning opportunities to gain greater ease in adjusting their visit flow, maximizing the information obtained from patients in a digital setting, and ensuring understanding on the part of patients and caregivers.

## Discussion

### Principal Findings

Telemedicine use in oncology, as characterized by the participants in this sample, reflected a complex interaction of multiple factors beyond pandemic-specific circumstances. A relative void of institutional steering and support allowed provider opinions about the relative benefit (eg, patient convenience or improved access) and risks (eg, concerns about compromising clinical care quality, impaired rapport building, and reduced communication quality) to drive variable use of telemedicine. A larger context of no clear oncologic *standard of care* pertaining to the efficacy and safety of telemedicine to fall back on further enabled a wide range of opinions and practices. These dynamics were skewed by technology challenges early in adoption, which led to preferential engagement with telephone over video for visit modality.

While there were clear positive impressions of telemedicine among staff and providers to support its ongoing use, at the time of this study, there were no significant continuing efforts or conversations among care teams or at a center- or department-wide level around long-term adaptation for sustained use. The presence of a local champion (an individual on work units who formally or informally promotes a process or intervention to their colleagues) is generally regarded as important to successful and sustained adoption of telemedicine [38,39] and is a core part of the “diffusion of innovation” model as put forth by Rogers and expanded upon by Greenhalgh et al [40]; such an individual was not apparent within the oncology department in our interviews. Study team members involved in telehealth deployment across this period noted that telemedicine champions seemed to already exist and emerge organically in other services at the organization; it is unclear precisely why this did not occur for oncology at our center, and a proactive effort to identify or designate a champion would be useful for future innovation efforts. Organizational learning and process improvement specific to telemedicine was slow to emerge, and expanded messaging and infrastructure in these areas could facilitate sustained, ongoing process improvement. Such approaches could provide an opportunity to revisit and shift some of the patterns set early in pandemic-forced adoption toward patient-oriented and shared goals (eg, minimizing frequency of patient transport when clinically feasible) and away from anecdotal impressions of care team members (eg, assumptions that certain patients or demographics are best served via face-to-face or telemedicine without directly eliciting preferences, or telemedicine use depending on provider comfort with technology rather than clinical context).

### Comparison With Prior Work

Organizational barriers may explain in part the differences in telemedicine use in our study versus the work by Patt et al [15], who reported less significant barriers in uptake and a >95% reported rate of video telemedicine use in a survey-based study; our study furthers theirs in that it used in-depth semistructured interviews rather than a survey tool to gather data for analysis.

Our findings align with larger theoretical frameworks around the implementation of novel processes and innovations, including the CFIR model as well as diffusion of innovation. These models all support the complex interplay of a myriad of factors influencing the success of any innovation and underscore the advantages of being able to plan and prepare for systemwide transitions such as this; such a preparation was not possible with the sudden shift in patient care necessitated by the COVID-19 pandemic. We noted the most influential factors pertaining to our rural, multisite cancer center above, including elements specific to the innovation itself (technologic challenges and the impacts of using telemedicine for the patient encounter), communication channels (a relative lack of leadership or operational support both in implementation and ongoing improvement work), and adopters (individual attitudes and motivations to adopt change).

### Limitations

While DCC serves a broad population base, most of the patients are located within Northern New England, and it is quite likely that other institutions with their own distinct populations and institutional cultures will have different challenges and opportunities. It is also possible that implementation in other circumstances without the rapid adoption due to a pandemic may have different dynamics and key factors for implementation. Our sample was sufficient to reach thematic saturation on major themes, but it leaves room for a more detailed exploration of some of the subthemes that emerged, including variation in provider messaging to patients around the visit options (in-person, telephonic, and video), provider and staff comfort with technology, and specific operational practices to minimize schedule disruption associated with telemedicine visits.

While staff and providers offered important insights to the attitudes, challenges, needs, and feedback of their patients, we did not directly interview patients for this study. It is notable that studies such as that carried out by Smith et al [28] included patients and caregivers in their interviews and found similar themes to our work regarding the acceptability and efficiency of telehealth generally for cancer care, alongside concerns regarding the lack of physical exam. Further investigation and analysis of patient perceptions of telemedicine use in cancer care—especially as we transition to a postpandemic environment where more patients are familiar with telemedicine and novelty—should further extend understanding of the risks and benefits of telemedicine use in oncology settings to equitably serve the needs of diverse populations.

### Conclusion

In a setting of decentralized care processes, early challenges in telemedicine implementation had an outsized impact on the nature and amount of sustained use. Proactively designed telemedicine care processes with attention to patient needs will be essential to supporting a sustained role for telemedicine in cancer care.

## Abbreviations

CFIR: Consolidated Framework for Implementation Research
DCC: Dartmouth Cancer Center
